# Supplementary material for: Spraying Small Water Droplets Acts as a Bacteriocide
Source: QRB Discov. 2020 Aug 6;1:e3. doi: 10.1017/qrd.2020.2 (PMC10392691; doi:10.1017/qrd.2020.2)
Supplement: Supplementary file 1 [file qrdsup.zip › S2633289220000022sup001.docx]

Supplementary Material

Spraying Small Water Droplets Acts as a Bacteriocide

**Authors:** Maria T. Dulay^1^†, Jae Kyoo Lee^1^†, Alison C. Mody^1^, Ramya Narasimhan^2^, Denise M. Monack^2^, Richard N. Zare^1^*

†These authors contributed equally to this work.

*Corresponding author. Email: [zare@stanford.edu](mailto:zare@stanford.edu)

**AquaROS disinfection of *E. coli* on LB agar plates**

*E. coli* colonies cultured on LB agar plates were sprayed directly with AquaROS at 6-cm spray distance, 120-psi nebulizing N_2_ or air, 20-min spray, and with/without 10-μL/min H_2_O flow (Fig. S1).


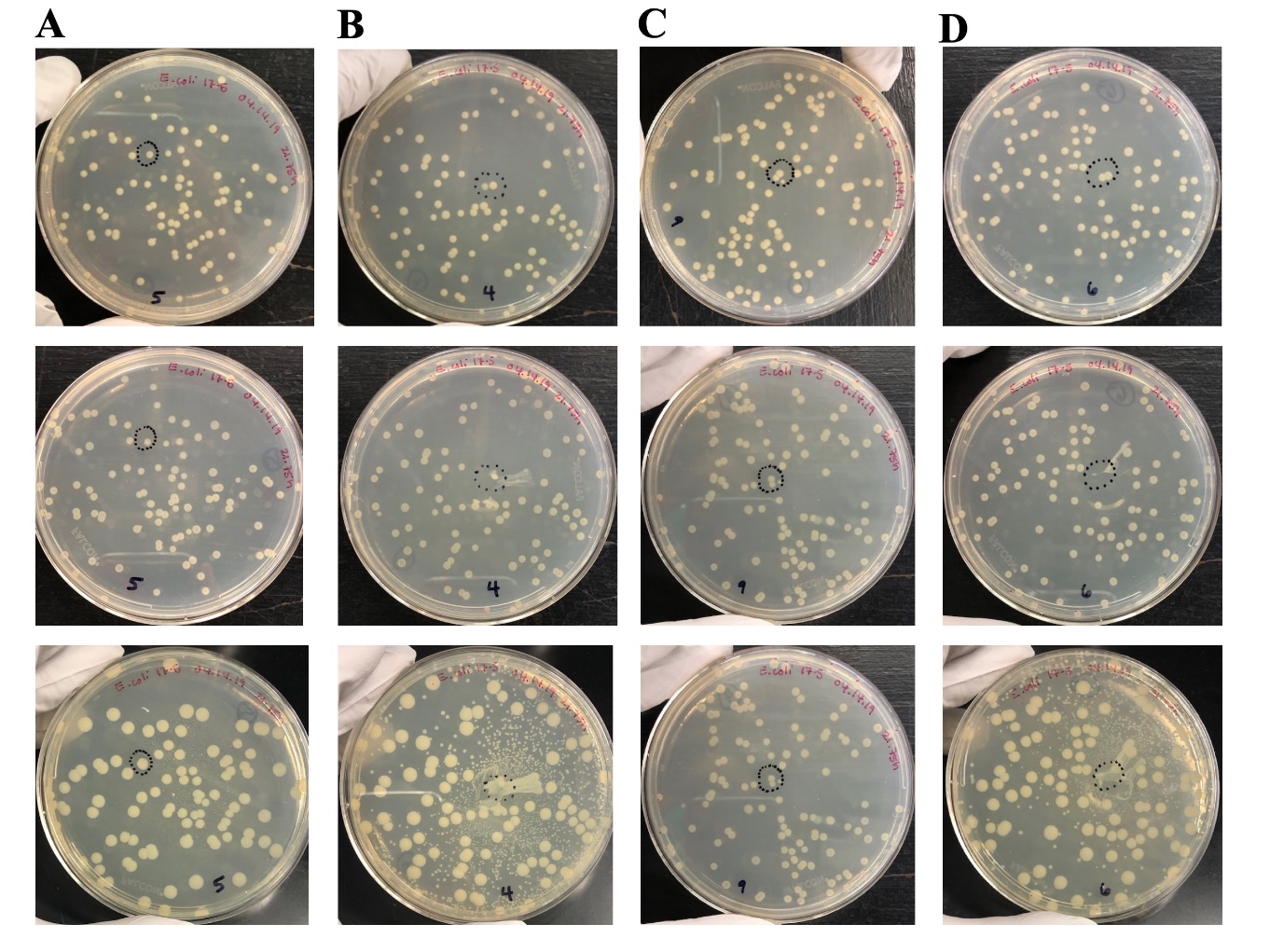


Before spray

After spray

After re-incubation

**Fig. S1.** Qualitative determination of *E. coli* dispersion during AquaROS spray. *E. coli* colonies on LB agar plates before AquaROS spray (top row). *E. coli* colonies cultured on LB agar plates after 20-min of AquaROS at 6-cm spray distance (middle row): (**A**) no H_2_O flow, (**B**) with 10 μL/min H_2_O flow with 120 psi of N_2_ nebulizing gas; and (**C**) no H_2_O flow and (**D**) with 10 μL/min H_2_O flow with 120 psi of air nebulizing gas. The treated plates were re-incubated at 37 °C for 18 h (bottom row). New colonies formed from the dispersion were observed in these plates. The sprayed area is marked with a black dashed circle. Red arrow shows the dispersion (“spreading out”) of bacteria during spray.

**Effect of high water flow rates**

*E.coli* colonies cultured on LB agar plates were exposed to AquaROS at two different water flow rates, 25 μL/min and 100 μL/min, at 9-cm spray distance and 120 psi N_2_ nebulizing gas. After the spray experiments (done in triplicates), bacterial cells were recovered. Fig. S2 is a plot of bacteria inactivation at each flow rate.


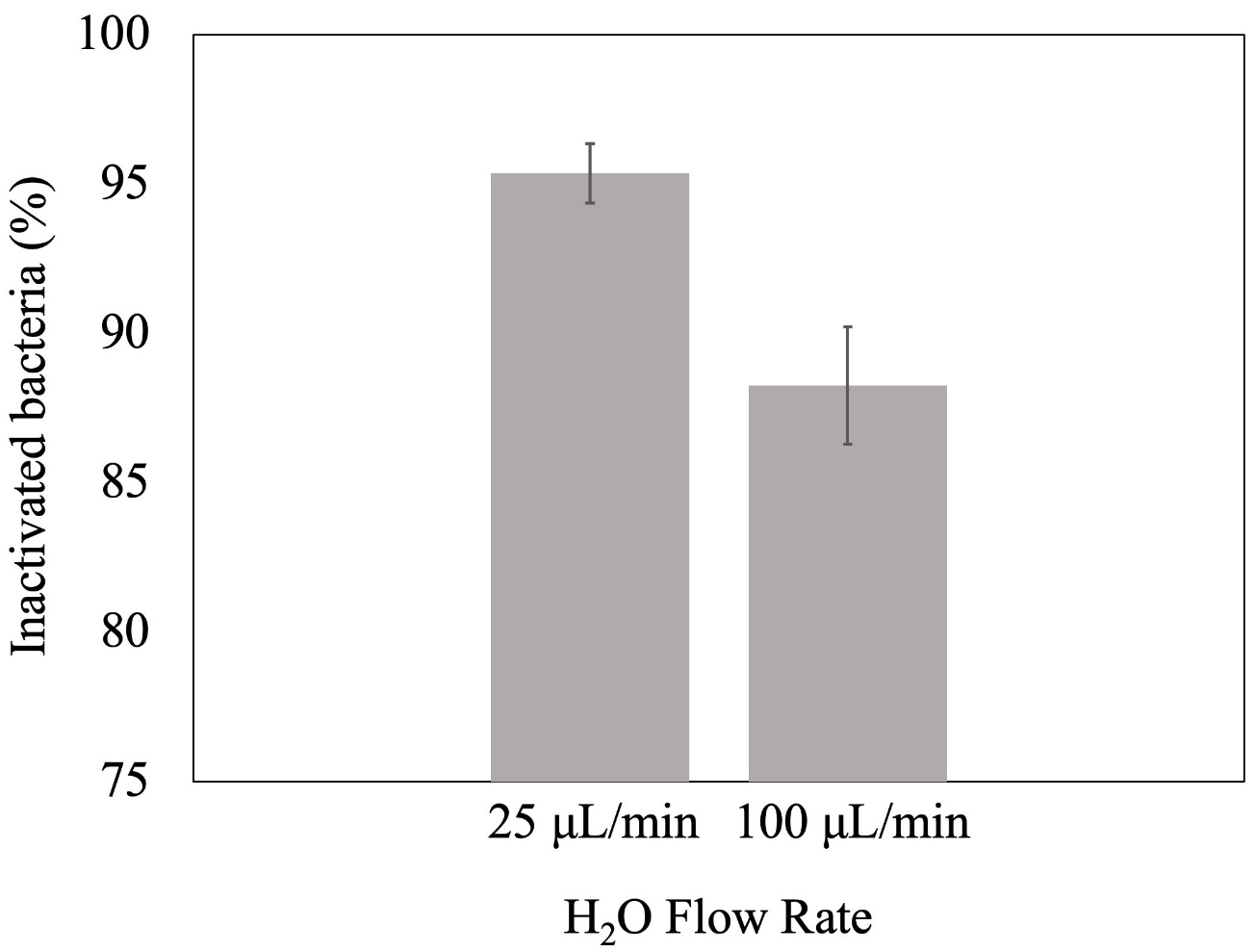


**Fig. S2.** Effect of H_2_O flow rate on bacteria inactivation. *E. coli* inoculum on stainless-steel disk (5 μL of 5 x 10^8^ CFU/mL, 5-min house vacuum-dried) sprayed with AquaROS generated with 25 μL/min and 100 μL/min HPLC-grade H_2_O flow rate with 120 psi N_2_ gas pressure, 9-cm spray distance, and 20-min spray time.

**Control experiments with single-capillary AquaROS spray**

The efficacy of AquaROS was tested with three different surfaces inoculated with *E. coli*: stainless-steel disk, plastic (Thermanox, 12-mm diameter, Ted Pella), and spinach leaf that was cut from a single leaf as a 1-cm x 1-cm square and washed 3X with distilled water and dried ambiently prior to *E. coli* deposition (Fig. S3). For these experiments, *E. coli* was cultured in LB broth at 37 °C for approximately 16 h. Using sterilized LB broth, the *E. coli* suspension was diluted to a concentration of 4.5 x 10^8^ CFU/mL, using a UV-vis spectrophotometer to monitor absorption at 600 nm. Infection of each surface was achieved by deposition of 10 μL *E. coli* suspension followed by house-vacuum drying in a desiccator for approximately 5 min. AquaROS was applied by spraying pure water from a single coaxial spray capillary with 120 psi N_2_ nebulizing gas and at a height of 1.5 cm from the *E. coli* surface and 10 μL/min flow rate for 20 min. Control samples were treated by depositing 100 μL of 3% H_2_O_2_ and allowing it to react for 20 min at room temperature, after which 1 mL PBS 1X (pH 7.4) was added to the glass vial containing the treated material, swirled, and pipetted into a 1-mL Eppendorf tube. The tube was centrifuged at 3,300 rpm for 5 min after which the supernatant was discarded and the pellet was resuspended before staining with SYTO 9 Green Fluorescent nucleic acid stain and propidium iodide (PI) for 15 min at room temperature and in the dark. SYTO 9 stains all dead and alive cells while PI stains dead cells. Cell death data was acquired by confocal fluorescence microscopy of each sample placed on a glass slide. Fig. S3 is a plot of *E. coli* death by AquaROS spray on the different surfaces compared to 3% H_2_O_2_. Over 90% of *E. coli* on stainless steel, plastic, and spinach leaf treated with AquaROS spray died, while approximately 60% of cells died when treated with 3% H_2_O_2_ and 15% with no treatment. Some cells are expected to die as a result of drying the samples under vacuum prior to disinfection experiments.


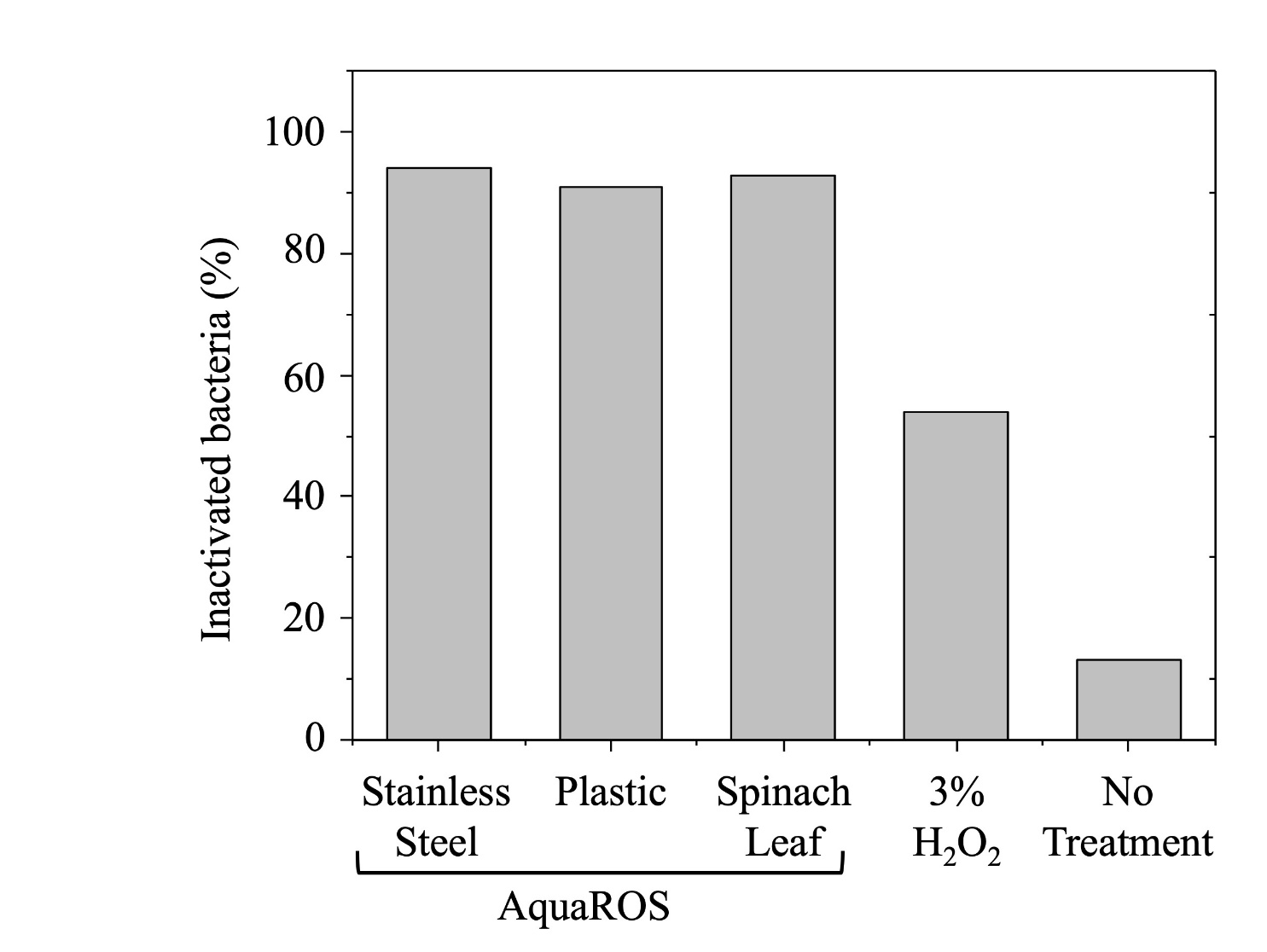


**Fig. S3.** Inactivation of *E. coli* on different surfaces and control experiments. Stainless steel disk, plastic, and spinach leaf were inoculated with *E. coli* and sprayed for 20 min with AquaROS, using a single-capillary coaxial sprayer, with a spray distance of 1.5 cm, 120 psi N_2_ nebulizing gas, 10 μL/min H_2_O flow. *E. coli* inoculated stainless-steel disk was disinfected by depositing 100 μL 3% H_2_O_2_ with 20-min disinfection time. All samples were processed with PI and SYTO 9 dye immediately after spraying for confocal fluorescence imaging.

**Disinfection of Spinach leaves**

Purchased spinach leaves were cut into 1-cm x 1-cm squares, which were sterilized by (1) rinsing under a gentle stream of tap water, (2) immersing in 1% bleach (v/v) for 20 min, and (3) rinsed with sterile biograde water. The leaves were allowed to dry in an area sterilized by the convective current of a Bunsen burner. Each sterilized piece was placed into a sterile Petri dish and inoculated with 5 μL *E. coli* suspension. Five minutes after bacteria deposition, the inoculated area was exposed to AquaROS for 20 min with an air compressor operated between 90 psi and 115psi and a spray distance of 9 cm. This experiment was done in triplicate. Non-sterilized spinach leaves were cut into 1-cm x 1-cm squares and treated with AquaROS for 20 min. This experiment was done once. Bacteria from each experiment was recovered by following the same protocol used with stainless steel disks. For the experiments using sterilized spinach leaves, colony counts before and after spray (Eq. 1) were used to determine viable bacteria count (Fig. S4). The weight of the spinach leaf before and after spray was used to calculate inactivation percentage of all microorganisms on the leaf.

**
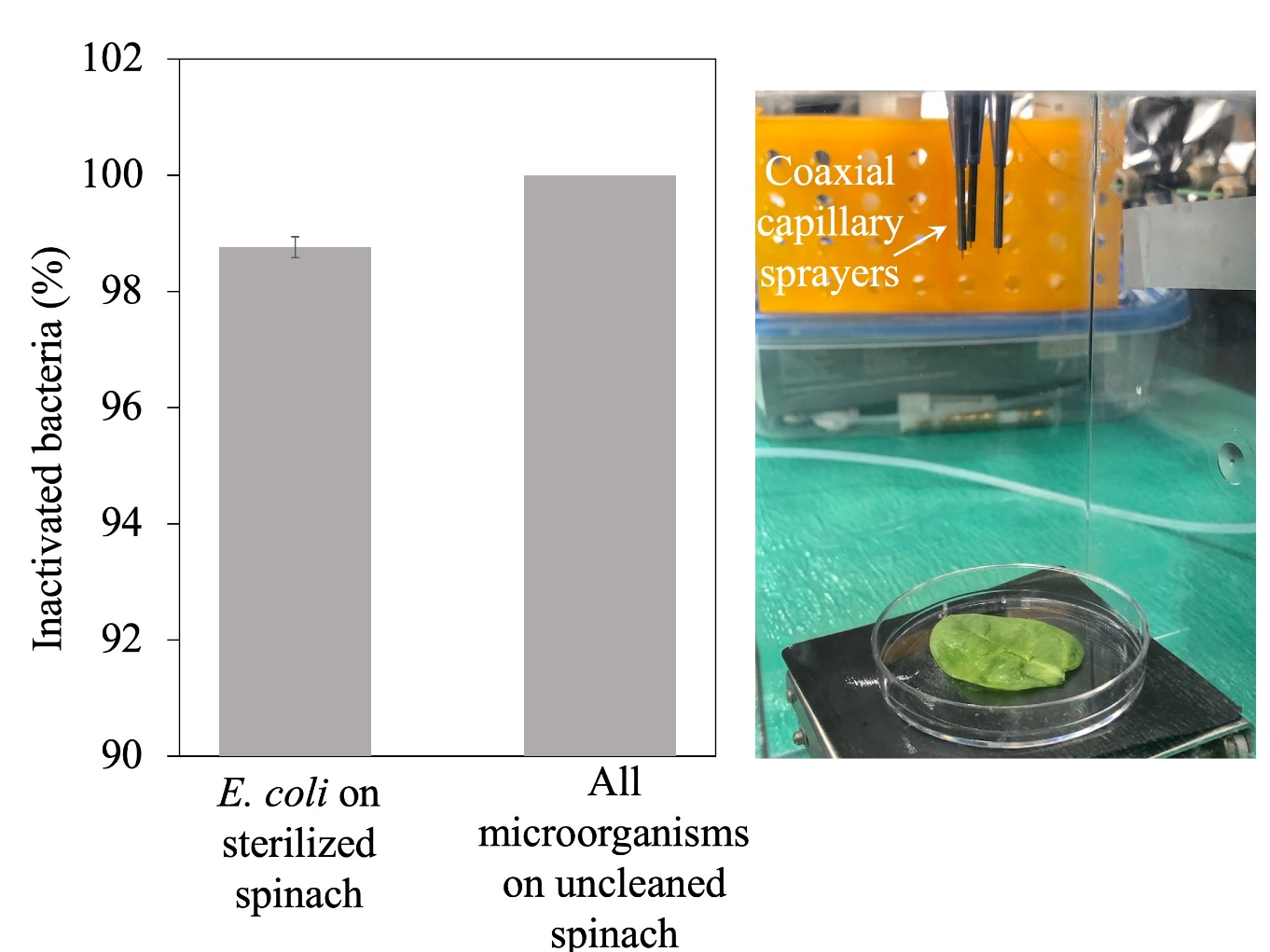
**

**Fig. S4.** AquaROS disinfection of spinach leaves. Sterilized spinach (1% bleach, 20 min) was inoculated with 5 μL *E. coli* suspension then sprayed 5 min later with AquaROS for 20 min, 9-cm spray distance and 120 psi N_2_ nebulizing gas. This was done in triplicate. An uncleaned spinach was sprayed directly with AquaROS under the same conditions as the sterilized spinach.

**Transmission electron microscopic analysis of AquaROS-treated *E. coli***


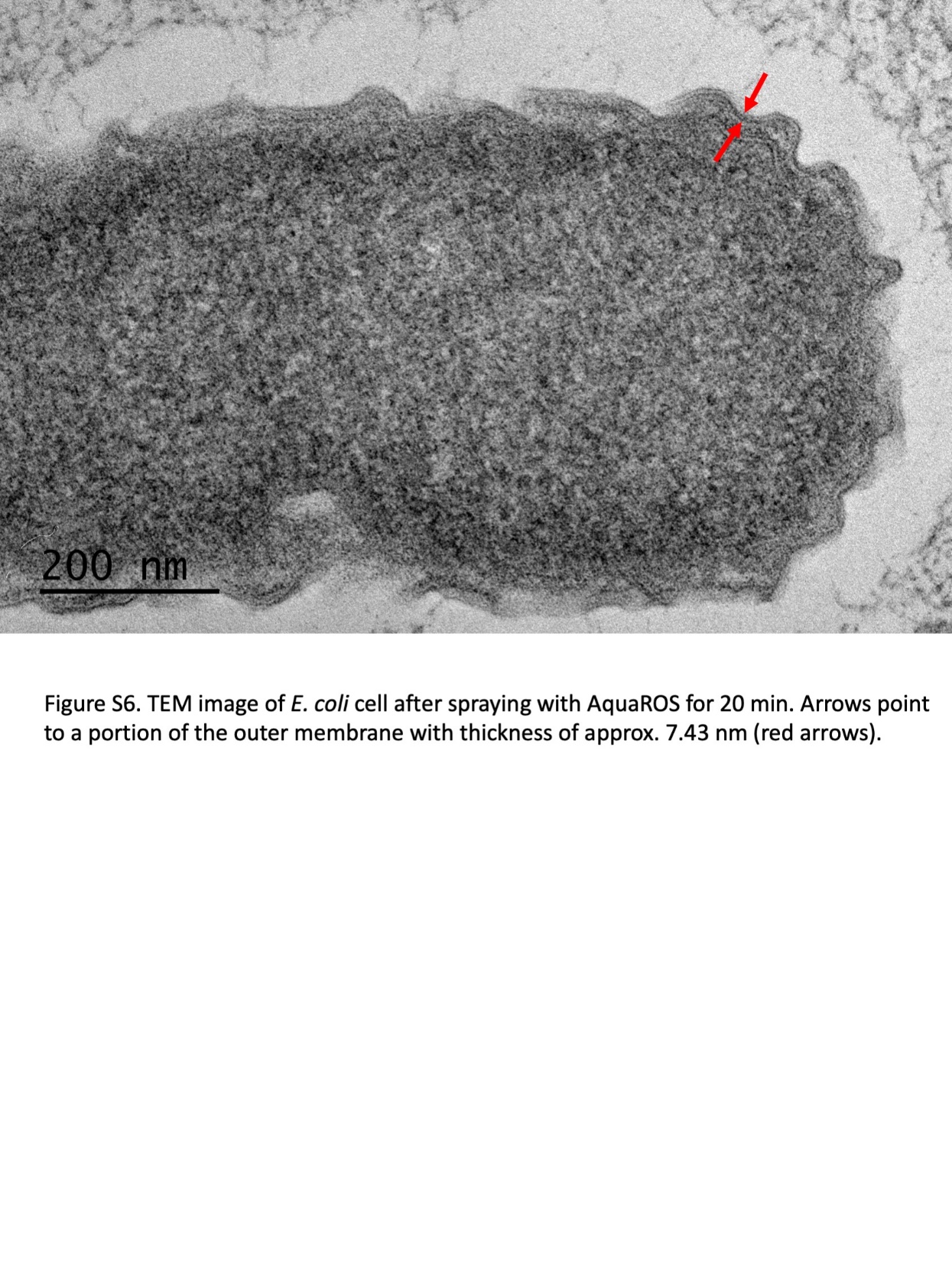


**Fig. S5.** TEM image of AquaROS-treated *E. coli* cell showing an outer membrane thickness of 6.9 ± 1.5 nm (red arrows).

**Tandem Mass Spectral identification of PG fragments after AquaROS spray**

Identity of phosphotidylglycerol (PG) fragments **4** were confirmed with tandem MS/MS and shown in Fig. S5. These fragments result from breaking of C⎯O bonds between the glycerol moiety and carbon chains.

**
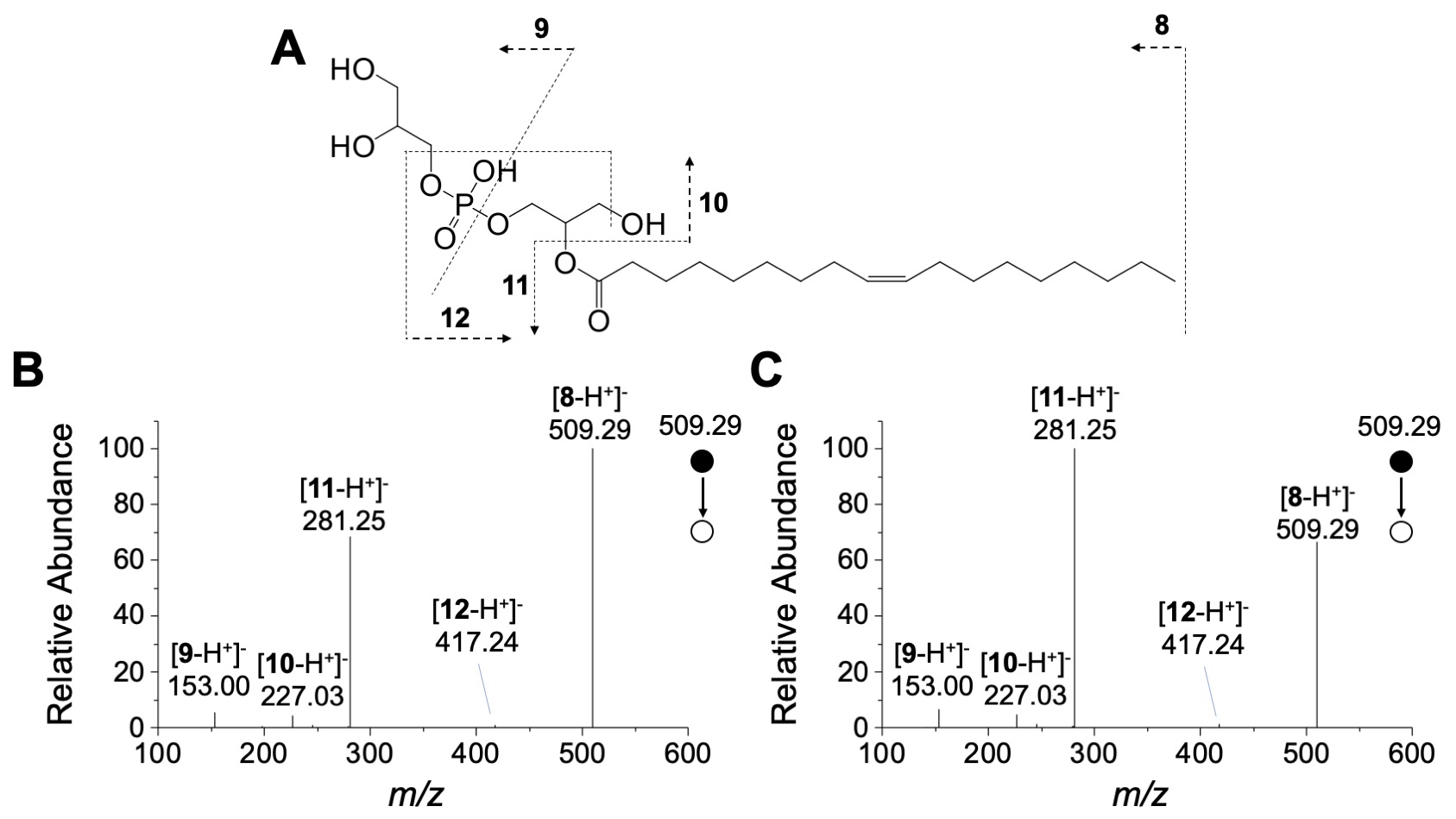
**

**Fig. S6.** Tandem mass spectrum (MS/MS) of PG fragmentation. (A) The identified structure **4** and its fragmentation pattern identified with tandem MS. (B) MS/MS spectrum of fragment **4** generated by AquaROS treatment. (C) MS/MS spectrum of standard sample.

To confirm that the loss of the carbon chain from PG was caused by chemical attack and not by drying or mechanical effects from the AquaROS spray, we compared the mass spectra of dried PG samples on glass slides (Fig. S6A) and treated with dry N_2_ gas that was used to nebulize bulk water in forming AquaROS (Fig. S6B). We observed little fragmentation in both cases as compared to AquaROS-treated samples.


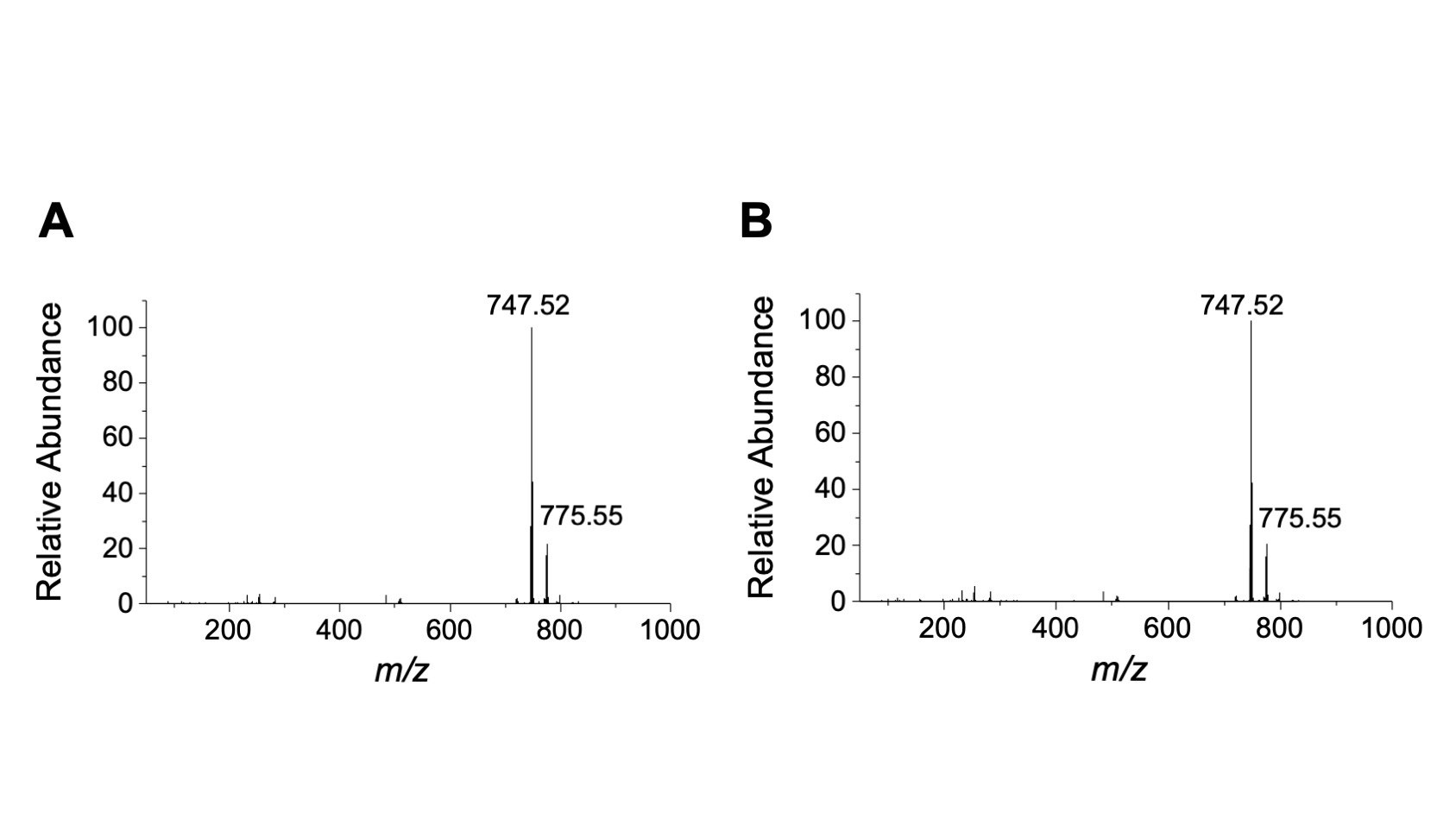


**Fig. S7.** Mass spectra of PG under different conditions. (A) PG molecules collected with drying for 20 minutes. (B) PG molecules treated only with N_2_ nebulizing gas for 20 minutes without AquaROS treatment.
